# GLP-1/GIP dual agonist tirzepatide in obstructive sleep apnea syndrome: mechanisms, evidence, and clinical perspectives

**DOI:** 10.3389/fmed.2026.1752341

**Published:** 2026-04-29

**Authors:** Qiujie Fang, Yipeng Zhu, Mei Rao, Peijun Liu

**Affiliations:** 1 Department of Respiratory and Critical Care Medicine, Ezhou Central Hospital, Ezhou, China; 2 Department of Pediatrics, The Central Hospital of Enshi Tujia and Miao Autonomous Prefecture, Enshi, China

**Keywords:** GLP-1/GIP dual agonist, metabolic regulation, obesity, obstructive sleep apnea, tirzepatide

## Abstract

Obstructive sleep apnea syndrome (OSAS) is closely associated with obesity and metabolic dysfunction. Tirzepatide, a dual GLP-1 and GIP receptor agonist, has demonstrated superior efficacy for weight reduction and improved metabolic health compared with single GLP-1 agonists. Emerging evidence indicates that tirzepatide may alleviate OSAS through its multifaceted effects on adiposity, inflammation, and neuromuscular regulation. This review synthesizes the pharmacological mechanisms, clinical findings, and safety data of tirzepatide in OSAS management. Preliminary studies show promising reductions in body weight, apnea–hypopnea index (AHI), and systemic inflammation, although long-term trials remain warranted. Further exploration into its integration with existing OSAS therapies could redefine the pharmacologic management of obesity-related OSAS.

## 1 Introduction

Obstructive sleep apnea syndrome (OSAS) is a chronic sleep-related breathing disorder characterized by recurrent upper airway collapse during sleep, leading to intermittent hypoxemia, sleep architecture disruption, and impaired daytime functioning (1, 2). OSAS has a high prevalence and significant adverse consequences. It is estimated that approximately 936 million adults worldwide suffer from moderate-to-severe OSAS, the vast majority of whom remain undiagnosed (3). With lifestyle changes and increasing obesity rates, the prevalence of OSAS continues to rise. In addition to profoundly compromising quality of life and daily functioning, this disorder contributes to the pathogenesis of numerous chronic diseases—including hypertension, coronary artery disease, stroke, diabetes, and cognitive impairment—and has been correlated with elevated all-cause mortality risk (4–6).

Obesity is among the most important modifiable risk factors for OSAS. Numerous studies have demonstrated that for every 10% increase in body mass index (BMI), the risk of developing OSAS increases by nearly six-fold (7, 8). Obesity contributes to the onset and progression of OSAS through multiple mechanisms. Cervical fat deposition narrows the upper airway and increases collapsibility, while abdominal fat accumulation limits diaphragmatic movement, reduces lung volume, and compromises airway stability under negative pressure. In addition, adipose tissue secretes proinflammatory cytokines, including tumor necrosis factor-*α* (TNF-α) and interleukin-6 (IL-6), which may impair neuromuscular control and exacerbate nocturnal respiratory instability (9, 10). Obesity-related metabolic disturbances, including metabolic syndrome, insulin resistance, and chronic low-grade inflammation, further contribute to OSAS pathogenesis. Standard therapies for OSAS include continuous positive airway pressure (CPAP), weight reduction, upper airway surgery, oral appliances, and neurostimulation. CPAP remains the most effective therapy for symptom control, but long-term adherence is poor, with 30–50% of patients unable to maintain regular use (11, 12). In this context, sustained weight-loss interventions, particularly pharmacologic approaches, have become an important therapeutic focus in obesity-related OSAS.

Glucagon-like peptide-1 (GLP-1) receptor agonists represent a class of pharmacological agents with both glucose-lowering and weight-reducing effects. Due to their remarkable ability to regulate metabolism, they have been widely adopted in the management of type 2 diabetes mellitus (T2DM) and obesity. In 2022, the FDA approved tirzepatide for the treatment of T2DM. Tirzepatide is a novel dual agonist that targets both the GLP-1 receptor and the glucose-dependent insulinotropic polypeptide (GIP) receptor (13, 14). Compared with conventional GLP-1 receptor agonists, such as liraglutide and semaglutide, tirzepatide has demonstrated superior efficacy in body weight control in several phase III clinical trials, with average weight reductions exceeding 20% in some study populations (15). In June 2024, the FDA officially approved tirzepatide for the treatment of obesity-associated OSAS, marking the first GLP-1–based therapy worldwide to receive this indication (16). This development marks an important advance in the pharmacological management of obesity-related OSAS. Through dual GLP-1 and GIP receptor activation, tirzepatide targets metabolic dysfunction while influencing anatomical and inflammatory factors associated with upper airway collapse (17, 18).

## 2 Literature search strategy

This narrative review aimed to synthesize current mechanistic and clinical evidence regarding tirzepatide in obesity-related OSAS. A comprehensive literature search was performed in PubMed, Web of Science, Embase, and Scopus from inception to October 2025 using the keywords “tirzepatide,” “GLP-1/GIP receptor agonist,” “obstructive sleep apnea,” and “obesity.” Priority was given to randomized controlled trials, major observational studies, and relevant mechanistic research. Only English-language publications were considered. The identified literature was narratively synthesized without formal study selection protocols, risk-of-bias assessment, or quantitative meta-analysis. A total of 112 articles were initially identified through comprehensive database searches. After screening titles and abstracts, 64 studies were selected for full-text review, and 41 articles were ultimately included in this review, including 12 randomized controlled trials, 16 observational studies, and 13 mechanistic or preclinical studies. Given the heterogeneity in study design, populations, intervention duration, and clinical endpoints, findings were interpreted with careful consideration of variability across the evidence base. Randomized trials, observational studies, and mechanistic investigations were assessed within their respective methodological contexts. Where discrepancies were observed, potential sources of variation—including patient characteristics, outcome measures, and follow-up duration—were qualitatively explored.

## 3 Pharmacological mechanisms of tirzepatide and its applications in metabolic diseases

Tirzepatide is a novel dual receptor agonist targeting both GLP-1 and GIP receptors. It exhibits a unique mechanism of action and remarkable metabolic benefits. Designed based on an in-depth understanding of incretin biology, tirzepatide can simultaneously activate GLP-1 and GIP receptors, thereby exerting synergistic effects that translate into superior outcomes in glycemic control, body weight management, and pancreatic *β*-cell function preservation (19, 20). GLP-1 is a gut-derived peptide hormone secreted by intestinal L cells. Its physiological actions include stimulating glucose-dependent insulin secretion, suppressing glucagon release, delaying gastric emptying, and enhancing satiety—mechanisms that collectively contribute to glucose lowering and weight reduction. GIP, on the other hand, is secreted by K cells of the small intestine. Although traditionally considered to have limited efficacy in patients with diabetes, recent studies indicate that, when acting synergistically with GLP-1, GIP enhances insulinotropic effects and improves overall metabolic homeostasis (20, 21). Structurally, tirzepatide is engineered to mimic the primary amino acid sequence of GIP while retaining partial agonistic activity at the GLP-1 receptor. This molecular configuration enables it to simultaneously engage and activate both incretin signaling pathways, thereby eliciting complementary and synergistic metabolic effects (22, 23). Through this dual-receptor mechanism, tirzepatide exerts more potent actions on glycemic regulation, appetite suppression, and energy balance than conventional single-pathway GLP-1 receptor agonists.

Evidence from the SURPASS phase III clinical trial program has consistently demonstrated the superior metabolic efficacy of tirzepatide compared with currently available GLP-1 receptor agonists, such as semaglutide. For instance, in the pivotal SURPASS-2 trial, patients receiving the highest tirzepatide dose (15 mg) achieved a mean body weight reduction of 11.2 kg, nearly twice that observed with semaglutide 1 mg (5.7 kg) (19). These findings underscore the enhanced therapeutic potential of dual incretin receptor activation in achieving robust and sustained metabolic improvements. The clinical application of tirzepatide in metabolic diseases has made substantial progress, particularly in the management of T2DM and obesity. Following its approval by the FDA in 2022 for the treatment of T2DM (19, 24). Tirzepatide has demonstrated consistent and clinically meaningful weight reduction across multiple studies, establishing it as one of the most promising non-surgical pharmacologic options for obesity management (15, 25). Beyond glycemic control and weight loss, tirzepatide exerts comprehensive effects on metabolic homeostasis, including improvements in insulin sensitivity, lipid metabolism, and inflammatory status.

**Figure 1:**
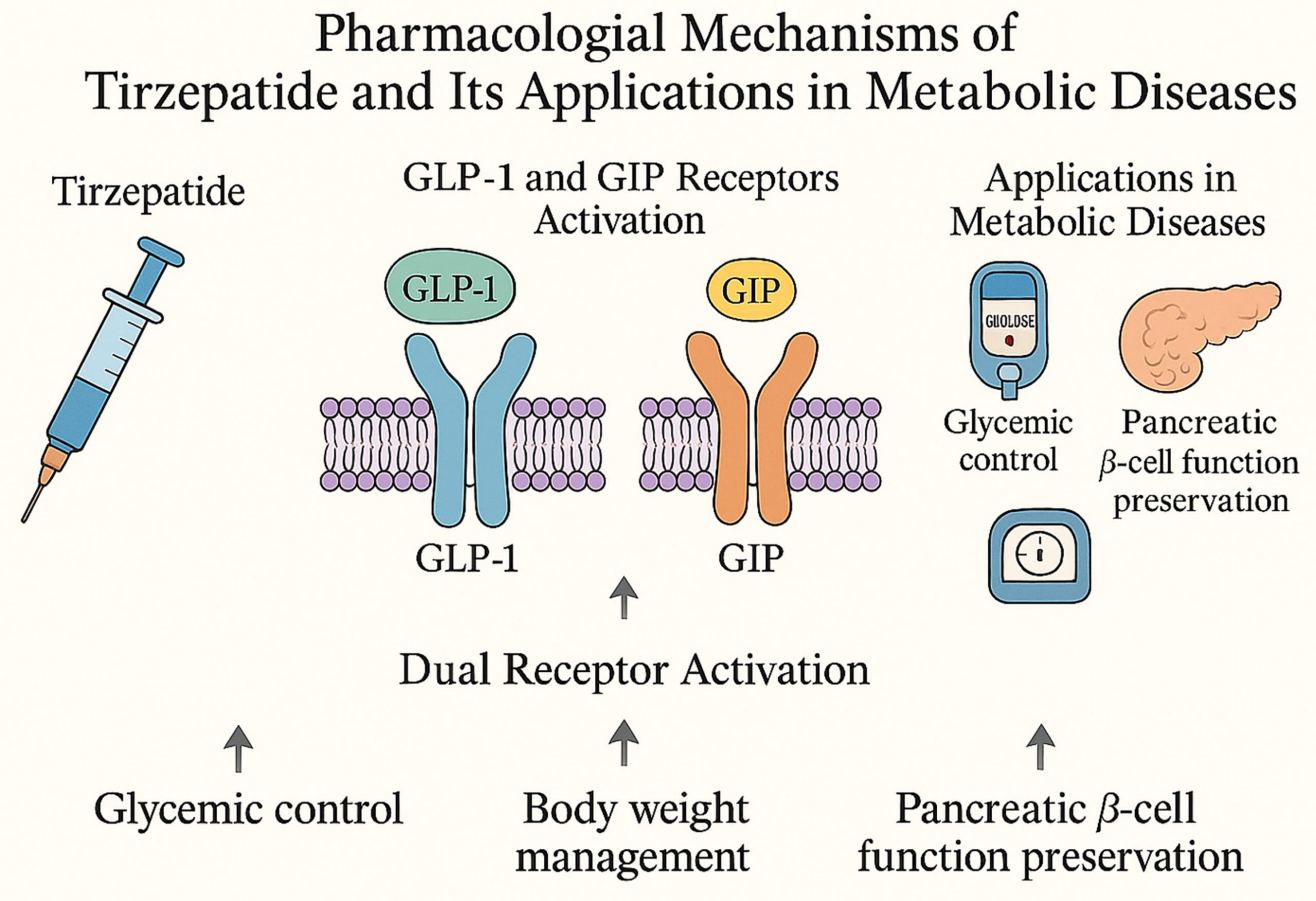
Pharmacological mechanisms of tirzepatide and its applications in metabolic diseases.

Given that obesity is a major modifiable risk factor for OSAS, and that the pathogenesis of OSAS involves metabolic dysregulation, systemic inflammation, and abnormal adipose tissue distribution, tirzepatide may offer multidimensional therapeutic benefits in this context (26). By improving overall metabolic health, reducing excess adiposity, and alleviating adipose tissue inflammation, tirzepatide could potentially mitigate upper airway collapsibility and ameliorate the underlying pathophysiological mechanisms of OSAS (27). Preliminary clinical evidence supports this hypothesis—studies in obese individuals treated with tirzepatide have shown a downward trend in the apnea–hypopnea index (AHI), suggesting that tirzepatide may have a beneficial effect on sleep-disordered breathing (28). Tirzepatide exerts its therapeutic effects by simultaneously activating GLP-1 and GIP receptors, leading to dual incretin pathway stimulation that enhances glycemic control, promotes weight reduction, and preserves pancreatic *β*-cell function (Figure 1).

## 4 Mechanisms of tirzepatide and its potential association with obstructive sleep apnea syndrome

### 4.1 Basic pharmacological mechanisms of tirzepatide

Tirzepatide is a novel dual receptor agonist that simultaneously activates the GLP-1 receptor and the GIP receptor. GLP-1 and GIP are incretin hormones secreted by intestinal epithelial cells following food intake. They enhance glucose-dependent insulin secretion, suppress glucagon release, delay gastric emptying, and reduce appetite, thereby contributing to glucose homeostasis and body weight regulation (29, 30). The phase 3 randomized withdrawal trial evaluated the long-term efficacy of tirzepatide for weight maintenance in adults with obesity or overweight. Continued tirzepatide therapy sustained and even augmented initial weight loss, whereas discontinuation resulted in marked weight regain, confirming its durable effectiveness and good tolerability for long-term weight management (31). Through dual pathway activation, tirzepatide improves glycemic control and induces substantial weight loss across clinical trials (15).

### 4.2 Tirzepatide in weight regulation and metabolic improvement

Obesity is one of the major risk factors for OSAS, and weight reduction has been associated with improvements in OSAS-related symptoms. Tirzepatide demonstrated remarkable weight-reducing efficacy in randomized controlled trials such as SURMOUNT-1 and SURMOUNT-2, with mean body-weight reductions ranging from 15 to 22.5% (32, 33). Its weight-loss effects are primarily achieved through several mechanisms, including central appetite regulation via hypothalamic pathways enhancing satiety, delayed gastric emptying that prolongs postprandial fullness, improvement of insulin sensitivity and metabolic homeostasis, and reduction of visceral and hepatic fat depots, which may alleviate respiratory mechanical burden and metabolic stress in OSAS (34).

### 4.3 Modulation of inflammatory status by tirzepatide

Beyond metabolic regulation, obesity-related OSAS is closely linked to chronic low-grade systemic inflammation driven by intermittent hypoxia. Recurrent nocturnal hypoxic episodes promote oxidative stress and inflammatory activation, characterized by elevated TNF-*α*, IL-6, and C-reactive protein levels (35). GLP-1 receptor agonists have been shown in multiple studies to exert anti-inflammatory effects, including inhibition of monocyte chemotaxis, suppression of macrophage inflammatory responses, and AMPK-mediated downregulation of the NF-κB signaling pathway, thereby reducing systemic inflammation (36). Similarly, GIP receptor activation has been reported to modulate adipose tissue immune responses and reduce inflammatory burden (37). Through these dual agonistic actions, tirzepatide may synergistically lower systemic inflammatory burden in OSAS patients, contributing to improved respiratory function and sleep quality.

### 4.4 Potential effects of tirzepatide on upper airway function

**Figure 2:**
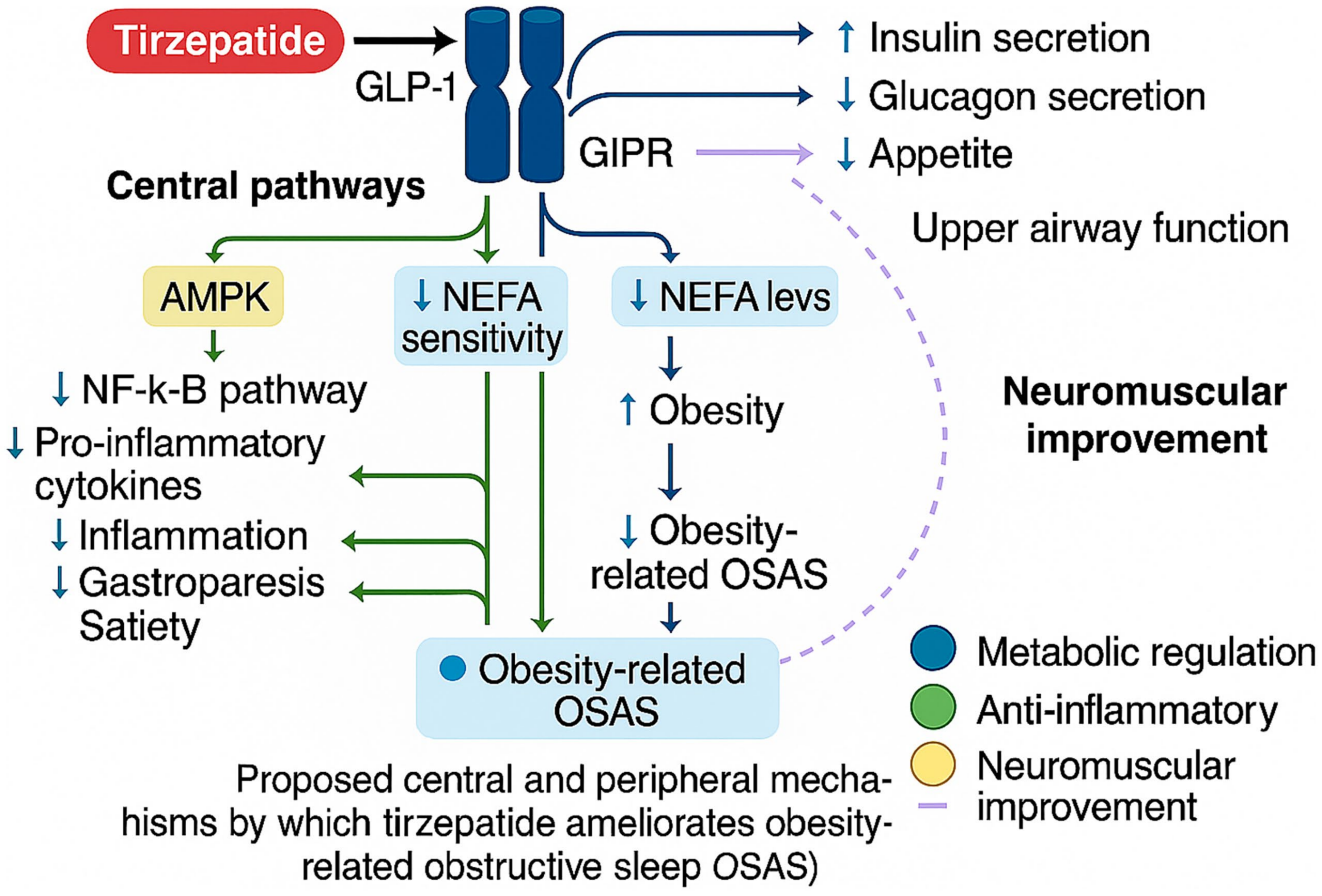
Proposed mechanisms by which tirzepatide ameliorates obesity-related obstructive sleep apnea syndrome.

In addition to metabolic and inflammatory modulation, emerging evidence suggests that incretin-based therapies may influence upper airway neuromuscular control. Experimental studies indicate that GLP-1 signaling can enhance genioglossus muscle tone, thereby reducing susceptibility to airway collapse during sleep (38, 39). An animal study demonstrated that GLP-1 enhances genioglossus muscle tone, thereby reducing the likelihood of upper airway collapse during sleep (40). GIP-related neuromodulatory effects have also been proposed, although respiratory-specific evidence remains limited (39, 41). Collectively, these findings suggest that tirzepatide may affect OSAS severity predominantly through metabolic and inflammatory improvement, with potential adjunctive neuromuscular contributions. However, direct clinical evidence for airway-specific effects remains scarce and warrants further investigation (42). In summary, tirzepatide modulates metabolic and inflammatory pathways that may enhance airway stability in obesity-related OSAS (Figure 2).

## 5 Clinical research progress and safety evaluation

### 5.1 Clinical efficacy studies

Tirzepatide, a novel dual GLP-1 and GIP receptor agonist, has demonstrated remarkable efficacy in the management of type 2 diabetes and obesity, as evidenced by multiple high-quality clinical trials (43, 44). With the growing understanding of the pathophysiological links between obesity and OSAS, the therapeutic potential of tirzepatide in OSAS—particularly among obese patients—has gained increasing attention. In 2023, a randomized, double-blind, placebo-controlled, multicenter trial investigated the efficacy of tirzepatide over 24 weeks in patients with moderate-to-severe obesity and OSAS. The tirzepatide group achieved a mean body-weight reduction of 18%, which appeared greater than that in the placebo group (45). Moreover, the AHI markedly decreased, accompanied by improvements in daytime sleepiness and sleep architecture normalization. This was the first study to systematically demonstrate that tirzepatide not only induces substantial weight loss but also shows improvements in sleep-disordered breathing among obese individuals. Beyond weight reduction and respiratory benefits, tirzepatide exerts favorable metabolic effects. Several studies have shown that tirzepatide reduces insulin resistance, improves glycemic control, and lowers inflammatory biomarkers, including C-reactive protein and IL-6 (46, 47). Its anti-inflammatory actions may help alleviate systemic inflammation and, indirectly, enhance upper airway neuromuscular stability, thus reducing airway collapsibility (48).

Importantly, tirzepatide may influence fat distribution, particularly by reducing visceral and cervical adiposity, which are closely associated with upper airway obstruction in OSAS. Decreasing neck and abdominal adiposity directly reduces mechanical airway resistance, a key factor in OSAS pathogenesis (38, 49). Obesity-associated adipose tissue secretes proinflammatory cytokines and adipokines—such as leptin and TNF-*α*—which negatively affect upper airway neuromuscular control and respiratory regulation (50). Tirzepatide mitigates these adverse effects by modulating cytokine expression, reducing airway inflammation, and restoring neuromuscular coordination, thereby promoting functional recovery of respiration at the molecular level (51). A recent multicenter retrospective study published in 2024 further validated tirzepatide’s real-world efficacy in OSAS management. Among over 500 obese patients with OSAS, long-term tirzepatide treatment led to significant reductions in body weight, AHI, and oxygen desaturation index, with particularly pronounced benefits in patients with concurrent glucose and lipid metabolism abnormalities (52). Additionally, tirzepatide improved quality-of-life scores and reduced the incidence of sleep-related cardiovascular events.

Although CPAP remains the standard first-line therapy for OSAS, the combination of tirzepatide and CPAP is emerging as a promising integrated treatment approach. Preliminary data suggest that tirzepatide, by improving weight and metabolic status, may enhance patient adherence to CPAP therapy and amplify overall therapeutic outcomes (53, 54). This integrative strategy could offer new avenues for managing treatment-resistant obesity-related OSAS. Nevertheless, the current evidence base for tirzepatide combined with CPAP remains limited, with short follow-up durations and small sample sizes. Further randomized controlled trials and long-term observational studies are needed to validate its efficacy and safety profile. Future research should also explore tirzepatide’s effects across different OSAS severities and phenotypes, paving the way for personalized therapeutic strategies.

### 5.2 Safety evaluation

In both clinical trials and real-world settings, tirzepatide has demonstrated a favorable safety and tolerability profile. The most commonly reported adverse events are gastrointestinal reactions, including nausea, vomiting, diarrhea, and constipation, which typically occur during the early phase of treatment and tend to diminish or resolve with continued therapy (55, 56). These symptoms are generally mild to moderate in severity and can be effectively managed by dose adjustment or extending the titration period (57).

With regard to the unique clinical context of OSAS, no evidence currently suggests that tirzepatide exacerbates sleep apnea symptoms or induces severe hypoxemic events (58). Preliminary safety analyses indicate that tirzepatide does not cause severe hypoglycemia and shows no adverse effects on central respiratory control (59). On the contrary, tirzepatide may help alleviate cardiopulmonary strain in OSAS patients by improving metabolic balance and systemic inflammation (54). Furthermore, tirzepatide has shown excellent cardiovascular safety. Data from the surpass trial series revealed that tirzepatide reduces the risk of major adverse cardiovascular events and improves blood pressure, lipid profiles, and inflammatory markers (60, 61). These cardiometabolic benefits are particularly relevant for OSAS patients with comorbid cardiovascular disease, potentially reducing the incidence of sleep-related cardiovascular complications.

**Figure 3:**
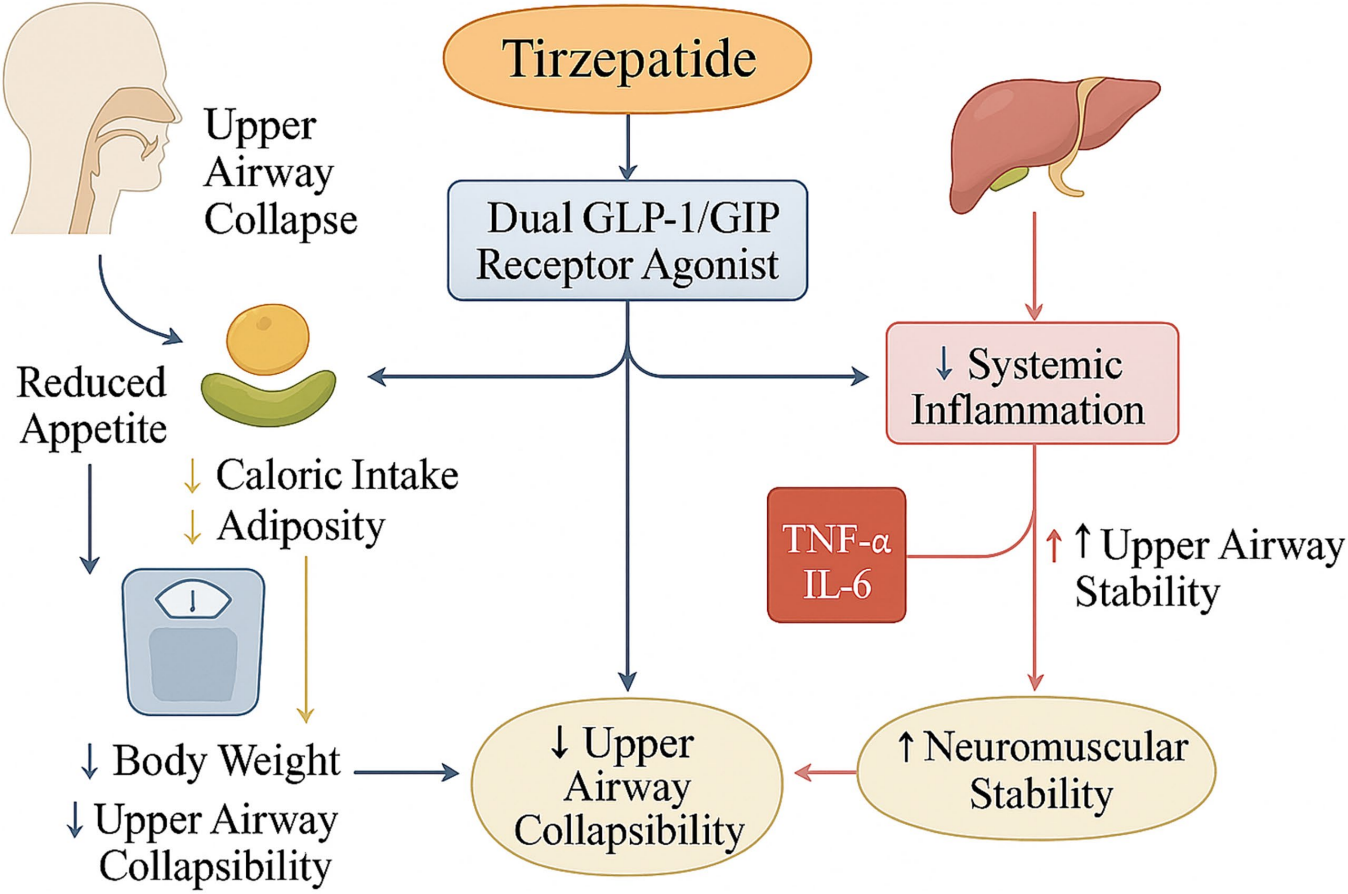
Mechanistic illustration of tirzepatide as a dual GLP-1 and GIP receptor agonist in improving obesity-related obstructive sleep apnea syndrome.

Despite its overall favorable safety profile, certain adverse reaction risks warrant attention. Gastrointestinal side effects may lead to treatment discontinuation in some patients, though severe cases remain rare and can be mitigated with appropriate monitoring (62). Considering that OSAS patients often present with multiple comorbidities—such as hypertension, coronary artery disease, and diabetes—further large-scale, long-term follow-up studies are needed to clarify tirzepatide’s safety in multimorbid populations (63, 64). Finally, it is important to highlight the immunomodulatory effects of tirzepatide. Although its anti-inflammatory properties may contribute to OSAS improvement, the complexity of immune regulation raises the possibility of unforeseen immune-related effects. The long-term impact on immune cell function and inflammatory microenvironment remains to be fully elucidated (65). Future research should focus on these aspects to ensure the sustained safety and efficacy of tirzepatide in clinical applications. The resulting weight reduction improves airway patency through decreased airway collapse and enhanced neuromuscular regulation, ultimately leading to improvement in OSAS and airway secretion function (Figure 3).

## 6 Conclusion

In summary, tirzepatide, a dual agonist of the GLP-1 and GIP receptors, shows promising potential for the management of obesity-related OSAS by promoting weight loss, improving metabolic function, and modulating inflammatory pathways. Tirzepatide targets key mechanisms involved in OSAS pathophysiology. Current clinical evidence suggests associations with reductions in body weight and AHI, along with improved glycemic control, although these benefits appear largely mediated by metabolic effects. Several studies have relatively short follow-up periods and limited sample sizes, which may constrain the evaluation of long-term efficacy and durability. However, given the novelty of this therapeutic approach, the available evidence may be influenced by publication bias and early optimism, and long-term mechanistic and clinical outcome data remain limited. Future large-scale randomized trials with extended follow-up and standardized respiratory endpoints are warranted to clarify durability and causal mechanisms.
